# Supplementary material for: Distinct ventral tegmental area neuronal ensembles are indispensable for reward-driven approach and stress-driven avoidance behaviors
Source: Nat Commun. 2025 Apr 2;16:3147. doi: 10.1038/s41467-025-58384-3 (PMC11965480; doi:10.1038/s41467-025-58384-3)
Supplement: Supplementary file 1 — Supplementary Information [file 41467_2025_58384_MOESM1_ESM.pdf]

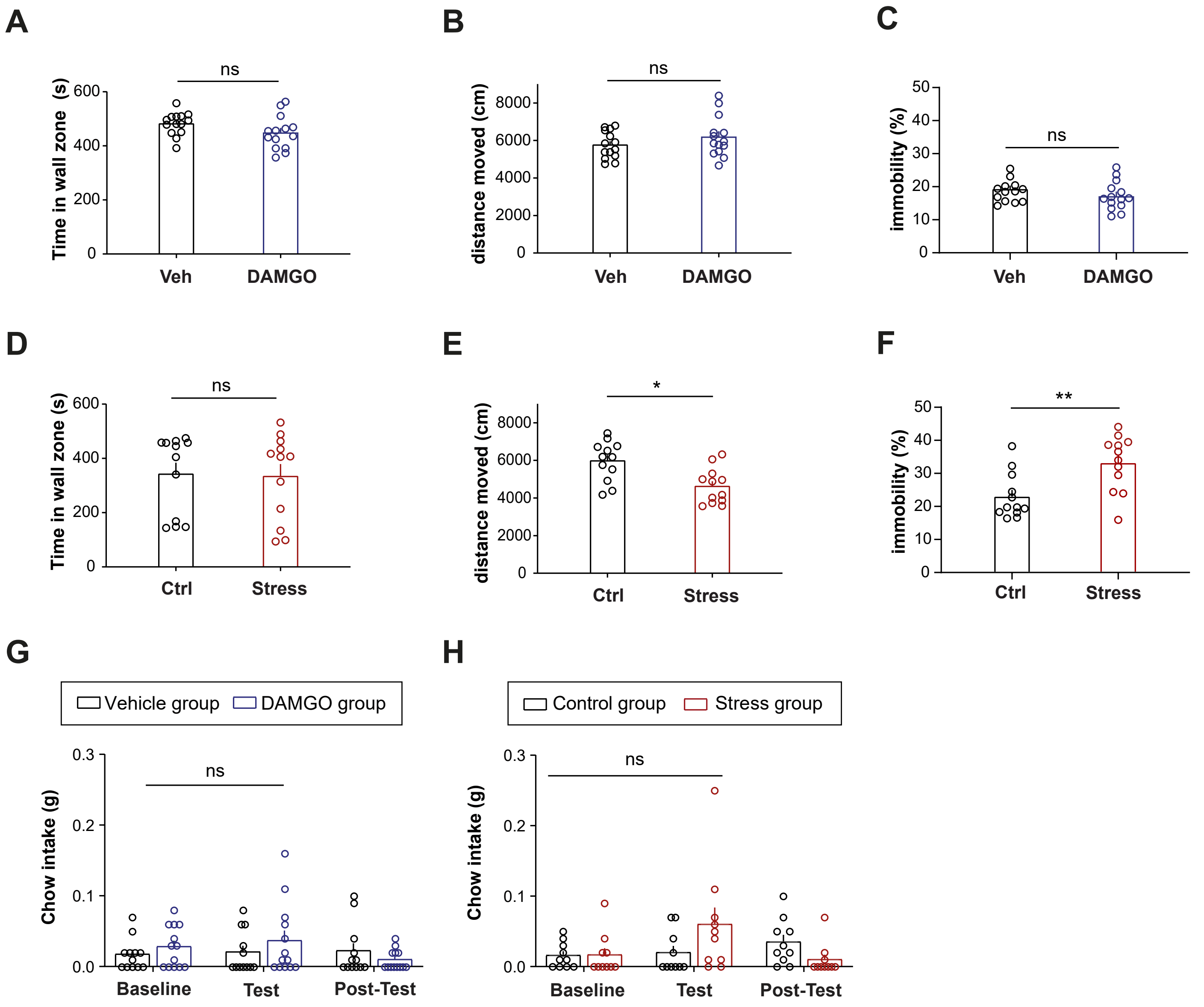

Supplementary Figure 1

SUPPLEMENTARY FIGURE 1 | Effect of stimuli of opposite valence on approach/avoidance behavior and related measures. (A) Amount of time spent in wall zone during the open field test after vehicle or DAMGO injection (N=14/group, unpaired t test, $t(26)=1.699$, $p=0.1012$). **(B)** Total distance moved during the open field test after vehicle or DAMGO injection (N=14/group, unpaired t test, $t(26)=1.21$, $p=0.2371$). **(C)** Proportion of time spent immobile during the open field test after vehicle or DAMGO injection (N=14/group, unpaired t test, $t(26)=1.31$, $p=0.2016$). **(D)** Amount of time spent in wall zone during the open field test after novel male C57BL/6J conspecific or acute stress exposure (N=12/group, unpaired t test, $t(22)=0.1433$, $p=0.8874$). **(E)** Total distance moved during the open field test after novel male C57BL/6J conspecific or social stress exposure (N=12/group, unpaired t test, $t(22)=3.318$, $p=0.0031$). **(F)** Proportion of time spent immobile during the open field test after novel male C57BL/6J conspecific or acute stress exposure (N=12/group, unpaired t test, $t(22)=3.26$, $p=0.0036$). **(G)** Amount of chow consumed during an 1-hour binge session after vehicle or DAMGO administration on test day ($N_{veh}=12$, $N_{DAMGO}=13$, Two-way RM ANOVA, Day x Ligand interaction $F(2,46)=1.604$, $p=0.2122$). **(H)** Amount of chow consumed during an 1-hour binge session after novel male C57BL/6J conspecific or acute stress exposure (N=10/group, Two-way RM ANOVA, Day x Group interaction, $F(2,36)=4.738$, $p=0.0149$, no significant post-hoc test). Data are presented as mean values + SEM. All statistical tests were performed two-sided. Source data are provided as a Source Data file.

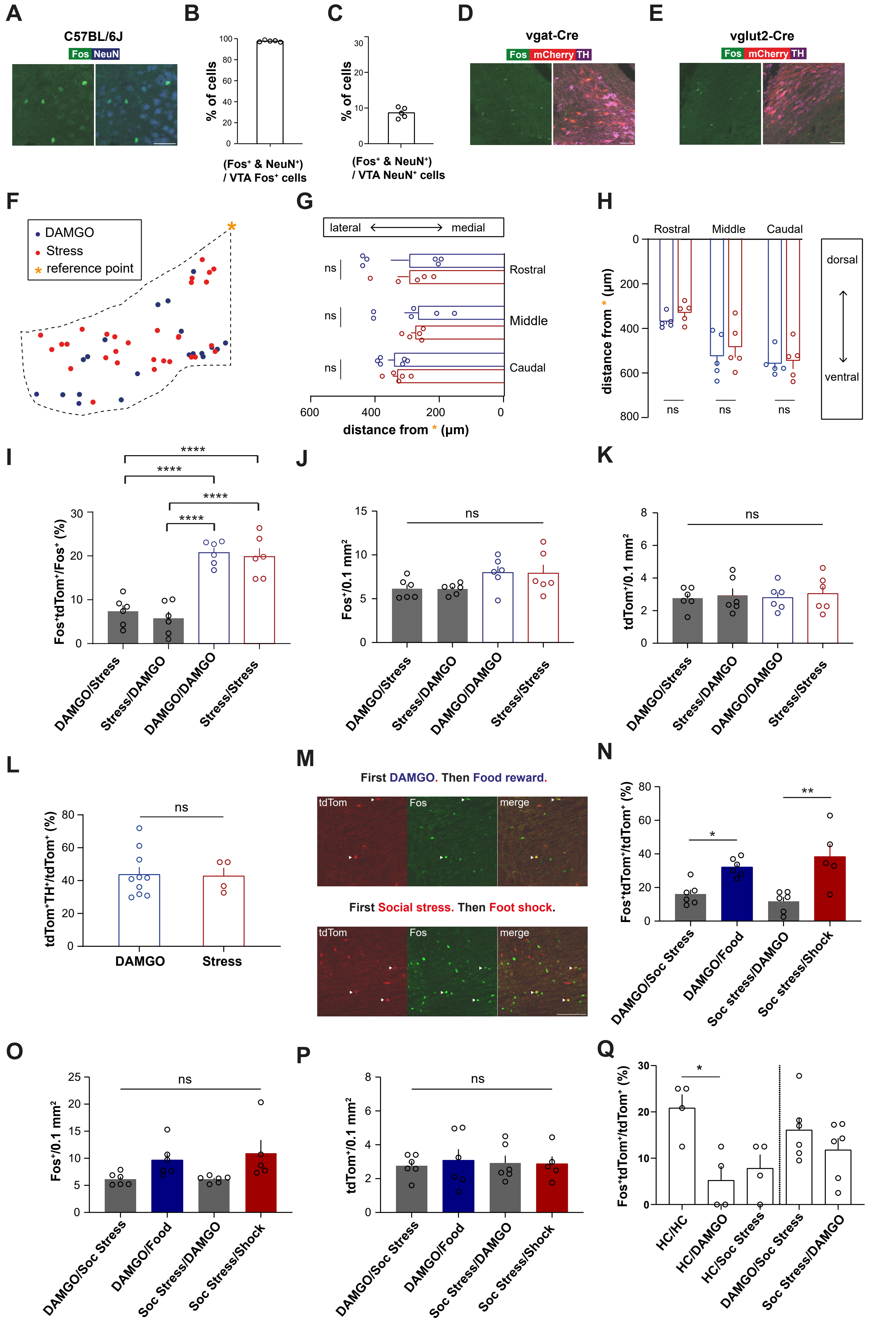

Supplementary Figure 2

SUPPLEMENTARY FIGURE 2 | Valence stimuli recruit distinct VTA ensembles. (A) Representative example of Fos/NeuN co-staining in the VTA. Scale bar: 50µm. **(B)** Percentage Fos^+NeuN^+/ Fos^+ (N=5 mice). **(C)** Percentage $Fos^+NeuN^+/NeuN^+$ (N=5 mice). **(D)** Representative example of Fos-mCherry-TH colabeling in the VTA for $VGAT^+$ cells. Scale bar: 50µm. **(E)** As (D) but for $Vglut2^+$ cells. **(F)** Representative examples of topography of Fos^+ cells in the VTA after DAMGO (blue) or social stress (red). **(G)** Mediolateral distance of Fos^+ neurons in the VTA from reference point after DAMGO or stress (N=5/group, unpaired t-test, rostral: $t(8)=0.00172$, $p=0.9987$, middle: $t(8)=0.1902$, $p=0.8539$, caudal: $t(8)=0.3951$, $p=0.7031$). **(H)** As (G) but dorsoventral distances (N=5/group, unpaired t-test, rostral: $t(8)=1.57$, $p=0.1550$, middle: $t(8)=0.6194$, $p=0.5529$, caudal: $t(8)=0.2889$, $p=0.78$). **(I)** Percentage $tdTom^+$ and Fos^+/Fos^+ cells (N=6/group, One-way ANOVA, $F(3,20)=28$, $p<0.0001$, Tukey's post-hoc comparisons: DAMGO/stress vs DAMGO/DAMGO, $q(20)=8.885$, $p<0.0001$, DAMGO/stress vs stress/stress, $q(20)=8.294$, $p<0.0001$, stress/DAMGO vs DAMGO/DAMGO, $q(20)=9.955$, $p<0.0001$, stress/DAMGO vs stress/stress, $q(20)=9.364$, $p<0.0001$). **(J)** Average number of $Fos^+/0.1\ mm^2$ (N=6/group, One-way ANOVA, $F(3,20)=2.462$, $p=0.0922$). **(K)** Average number of $tdTom^+/0.1\ mm^2$ (N=6/group, One-way ANOVA, $F(3,20)=0.1122$, $p=0.9519$). **(L)** Percentage $tdTom^+TH^+/tdTom^+$ for VTA_{DAMGO} and VTA_{STRESS} ($N_{DAMGO}=10$, $N_{STRESS}=4$, unpaired t test, $t(12)=0.1231$, $p=0.9041$). **(M)** Representative images of VTA slices from TRAP2xAi14 animals expressing tdTomato in DAMGO- or stress-activated neurons (TRAP) and Fos in food reward- or foot shock stress-activated neurons during a second exposure. White arrowheads: co-localized tdTomato (TRAP) and Fos. Scale bar: 50µm. **(N)** Percentage $tdTom^+$ and $Fos^+/tdTom^+$ cells ($N_{D/S}=6$, $N_{D/FR}=6$, $N_{S/D}=6$, $N_{S/FS}=5$, One-way ANOVA, $F(3,19)=9.471$, $p=0.0004$, Tukey's post-hoc comparisons: DAMGO/social (soc) stress vs DAMGO/food reward, $q(19)=4.097$, $p=0.0421$, stress/DAMGO vs stress/foot shock, $q(19)=6.439$, $p=0.0011$. **(O)** Number of $Fos^+/0.1\ mm^2$ ($N_{D/S}=6$, $N_{D/FR}=6$, $N_{S/D}=6$, $N_{S/FS}=5$, One-way ANOVA, $F(3,19)=3.739$, $p=0.0288$). Tukey's post-hoc comparisons: No significant comparisons. **(P)** Number of $tdTom^+/0.1\ mm^2$ ($N_{D/S}=6$, $N_{D/FR}=6$, $N_{S/D}=6$, $N_{S/FS}=5$, One-way ANOVA, $F(3,19)=0.07999$, $p=0.9701$). **(Q)** Percentage $tdTom^+$ and $Fos^+/tdTom^+$ cells ($N_{HC/HC}=4$, $N_{HC/D}=4$, $N_{HC/S}=4$, $N_{D/S}=6$, $N_{S/D}=6$, One-way ANOVA, $F(4,19)=4.209$, $p=0.0132$, Tukey's post-hoc comparisons: HC/HC vs HC/DAMGO, $q(19)=4.998$, $p=0.0167$). Last two conditions reshown from Fig. 2H for ease of comparison. Data are presented as mean values + SEM. All statistical tests were performed two-sided. Source data are provided as a Source Data file.

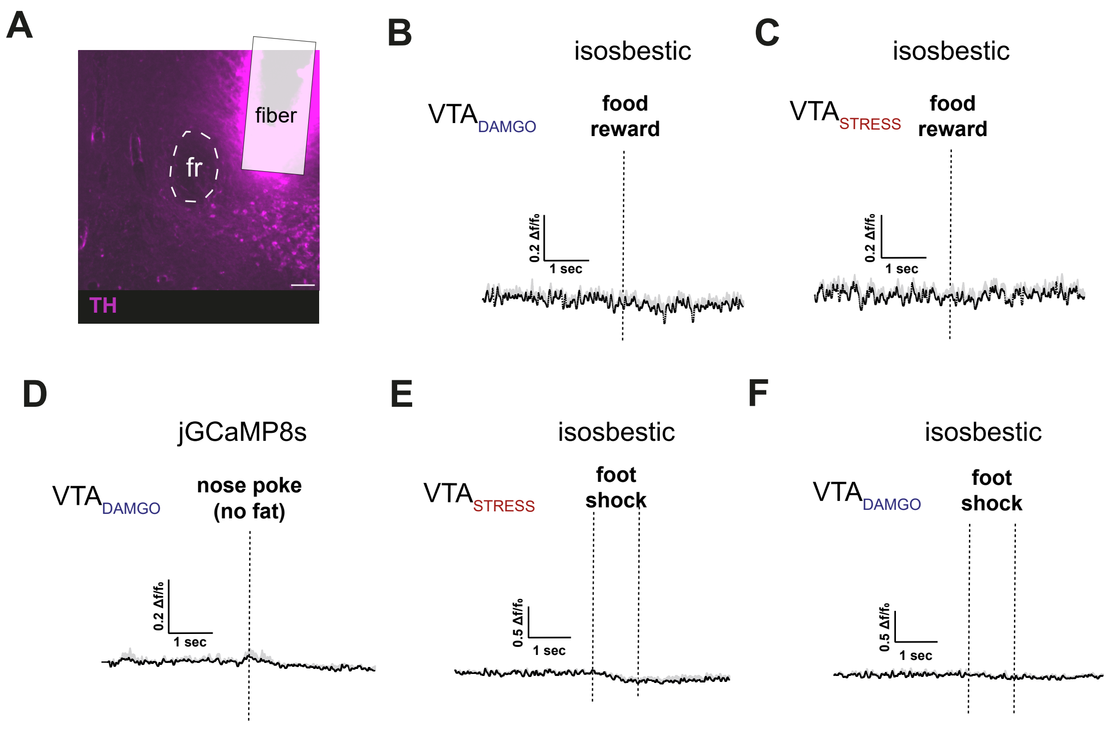

Supplementary Figure 3

SUPPLEMENTARY FIGURE 3 | Control parameters for VTA_{DAMGO} and VTA_{STRESS} ensemble in vivo responses. **(A)** Immunohistochemistry against TH and fiber trace over the VTA. Scale bar 100µm. fr fasciculus retroflexus **(B)** Line plot showing the average $\Delta f/F_0$ of isosbestic control for VTA_{DAMGO} ensemble during food reward. **(C)** Line plot showing the average $\Delta f/F_0$ of isosbestic control for VTA_{STRESS} ensemble during food reward. **(D)** Line plot showing the average $\Delta f/F_0$ jGCaMP8s signal from VTA_{DAMGO} time-locked to nose-poking in an empty port. **(E)** Line plot showing the average $\Delta f/F_0$ of isosbestic control for VTA_{STRESS} ensemble during foot shock. **(F)** Line plot showing the average $\Delta f/F_0$ of isosbestic control for VTA_{DAMGO} ensemble during foot shock. Data are presented as mean values + SEM. All statistical tests were performed two-sided. Source data are provided as a Source Data file.

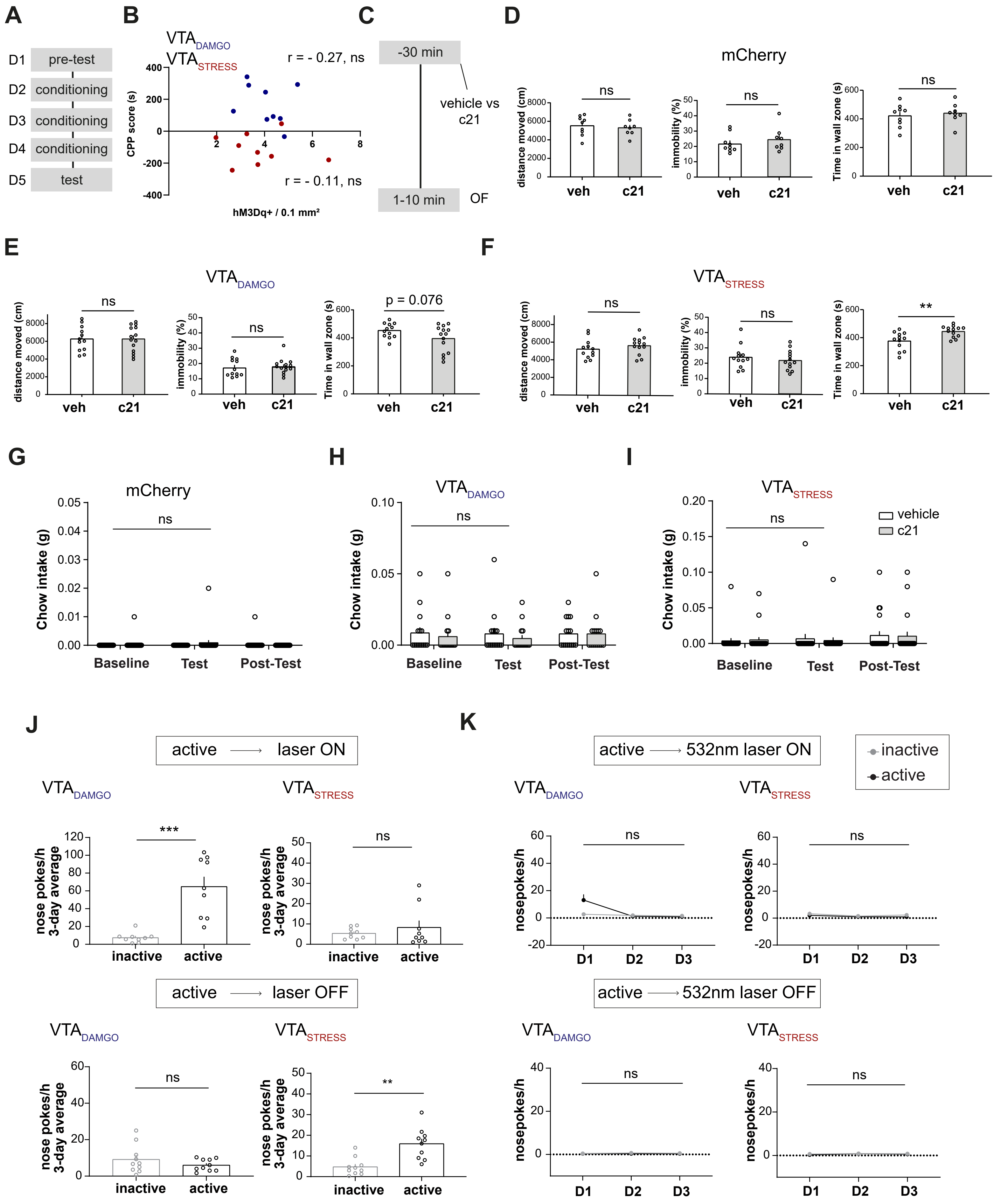

Supplementary Figure 4

SUPPLEMENTARY FIGURE 4 | Control measures for stimulation of VTA_{DAMGO} or VTA_{STRESS} ensembles. **(A)** 5-day CPP experimental timeline. **(B)** Correlation between ensemble size (number of hM3Dq-expressing neurons) and CPP score for VTA_{DAMGO} and VTA_{STRESS} groups (N_{DAMGO}=9, Pearson's r=-0.2728, p=0.4776. N_{STRESS}=9, Pearson's r=0.1086, p=0.7809). **(C)** Open Field experimental timeline**.** **(D)** Distance moved (left), %immobility (middle) and time near wall (right) in the open field for mCherry mice, after vehicle or c21 in injection (N_{veh}=9, N_{c21}=8, distance: unpaired t test, t(15)=0.4842, p=0.6352, immobility: unpaired t test, t(15)=0.8293, p=0.4199, wall zone: unpaired t test, t(15)=0.4876, p=0.6329). **(E)** As (D), but for the VTA_{DAMGO} group (N_{veh}=12, N_{c21}=14, distance moved: unpaired t test, t(24)=0.0003532, p=0.09997, immobility: unpaired t test, t(24)=0.3619, p=0.7206, wall zone: unpaired t test, t(24)=1.854, p=0.0761). **(F)** As (D), but for the VTA_{STRESS} group (N_{veh}=12, N_{c21}=13, distance: unpaired t test, t(23)=1.021, p=0.3181, immobility: unpaired t test, t(23)=0.6430, p=0.5266, wall zone: unpaired t test, t(23)=3.206, p=0.0039). **(G)** Chow intake during baseline, test, and post-test days for mCherry group (N=22, Two-way RM ANOVA, Day x Ligand interaction, F(2,84)=0.2749, p=0.7603). **(H)** As (G), but for VTA_{DAMGO} group (N=15, Two-way RM ANOVA, Day x Ligand interaction, F(2,56)=0.1318, p=0.8768). **(I)** As (G), but for VTA_{STRESS} group (N=21, Two-way RM ANOVA, Day x Ligand interaction, F(2,80)=0.1651, p=0.8481). **(J)** Top: Number (3d average) of nose-pokes to activate a 473nm laser for VTA_{DAMGO} and VTA_{STRESS} groups (VTA_{DAMGO} : N=9, paired t test, t(8)=5.622, p=0.0005. VTA_{STRESS}: N=10, paired t test, t(9)=1.446, p=0.1821). Bottom: As top, but to deactivate the 473nm laser (VTA_{DAMGO} : N=9, paired t test, t(8)=0.8623, p=0.4136. VTA_{STRESS}: N=10, paired t test, t(9)=4.104, p=0.0027). **(K)** Top: Number of nose pokes to activate a 532 nm laser for VTA_{DAMGO} and VTA_{STRESS} on D1-D3 (N_{DAMGO}=9, Two-way RM ANOVA, Day x Nose-poke interaction, F(2,32)=6.434, p=0.0045, no significant post-hoc. N_{STRESS}=10, Two-way RM ANOVA, Day x Nose-poke interaction, F(2,36)=0.2363, p=0.7908). Bottom: As top, but to deactivate a 532 nm laser (N_{DAMGO}=9, Two-way RM ANOVA, Day x Nose-poke interaction, F(2,32)=0.1231, p=0.8846. N_{STRESS}=10, Two-way RM ANOVA, F(2,36)=0.1667, p=0.8471). Data are presented as mean values + SEM. All statistical tests were performed two-sided. Source data are provided as a Source Data file.

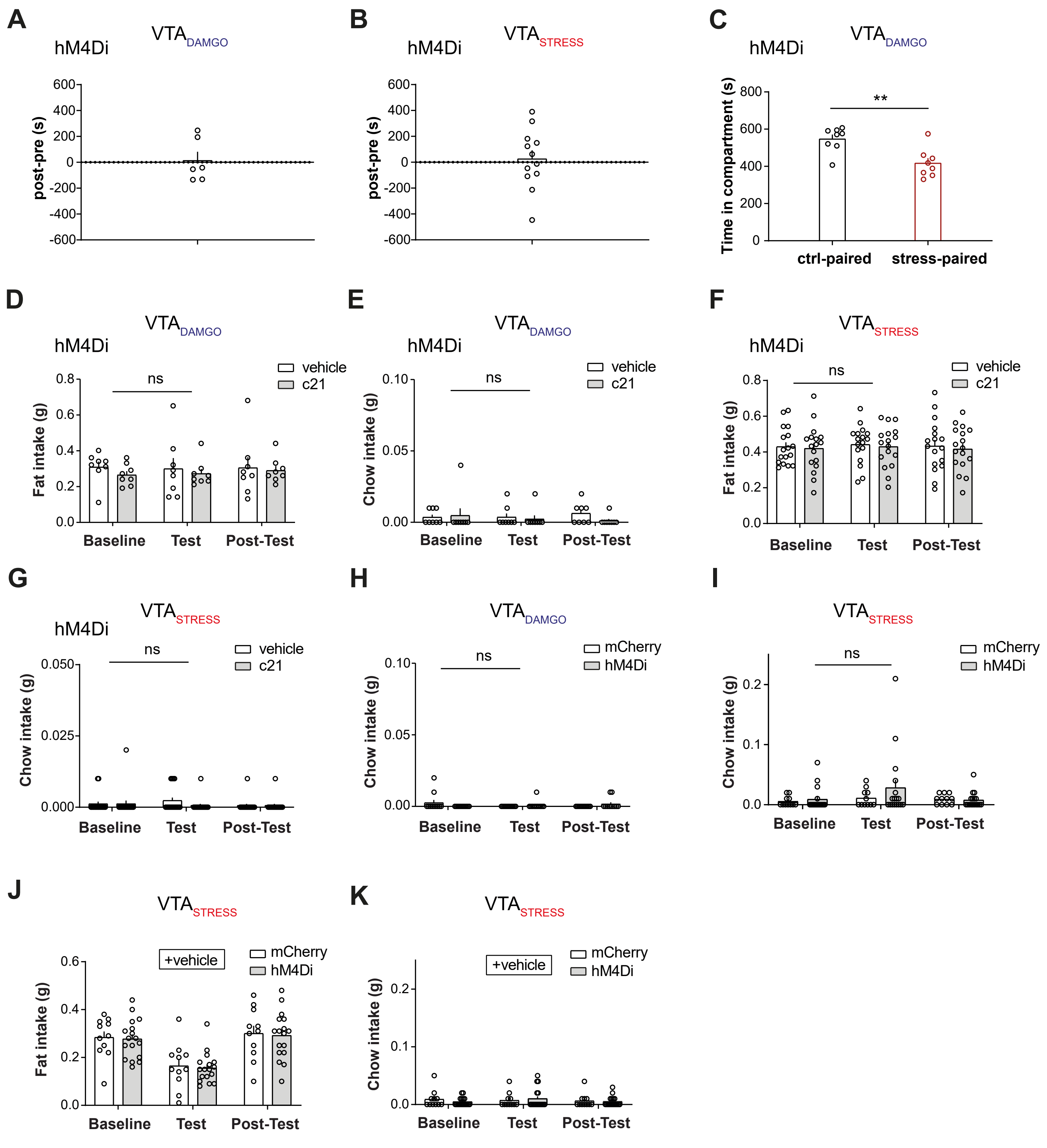

Supplementary Figure 5

SUPPLEMENTARY FIGURE 5 | Control measures for inhibition of VTA_{DAMGO} or VTA_{STRESS} ensembles. **(A)** Preference score for VTA_{DAMGO} hM4Di-expressing mice (N=6, paired t test, t(5)=0.1963, p=0.8521). **(B)** Preference score for VTA_{STRESS} hM4Di-expressing mice (N=12, paired t test, t(11)=0.3854, p=0.7073). **(C)** Time spent in control-paired or stress-paired compartments in VTA_{DAMGO} hM4Di-expressing animals (N=8, paired t test, t(14)=3.599, p=0.0029). **(D)** Amount of fat consumed for VTA_{DAMGO} hM4Di-expressing animals during baseline, test, and post-test days. (N=8, Two-way RM ANOVA, Day x Ligand interaction, F(2,28)=0.1662, p=0.8477). **(E)** As (D) but for chow consumed (N=8, Two-way RM ANOVA, Day x Ligand interaction, F(2,28)=0.6488, p=0.5304). **(F)** Fat consumed for VTA_{STRESS} hM4Di-expressing animals during baseline, test, and post-test days (N=17, Two-way RM ANOVA, Day x Group interaction, F(2,64)=0.008964, p=0.9911). **(G)** As (F) but for chow consumed (N=17, Two-way RM ANOVA, Day x Group interaction, F(2,64)=0.8889, p=0.4161). **(H)** Chow consumed during baseline, test, and post-test day for VTA_{DAMGO} mCherry- or hM4Di-expressing mice. During test day mice received an injection of c21 (2 mg/kg) followed by an injection of DAMGO (1 mg/kg) before the beginning of the feeding session ($N_{mCherry}$=11, N_{hM4Di}=11, Day x Group interaction, Two-way RM ANOVA, F(2,40)=3.231, p=0.05). **(I)** Chow consumed during baseline, test, and post-test day for VTA_{STRESS} mCherry- or hM4Di-expressing mice. During test day mice received an injection of c21 (2 mg/kg) followed by a 20 sec episode of social stress before the beginning of the feeding session ($N_{mCherry}$=11, N_{hM4Di}=17, Two-way RM ANOVA, Day x Group interaction, F(2,52)=0.9741, p=0.3843). **(J)** Fat consumed during baseline, test, and post-test days for VTA_{STRESS} mCherry- and hM4Di-expressing mice. During test day mice received a vehicle injection followed by a 20 s episode of social stress prior to the beginning of the feeding session ($N_{mCherry}$=11, N_{hM4Di}=17, Two-way RM ANOVA, Day main effect, F(1.818,47.27)=36.35, p<0.0001). **(K)** As (J), but for chow intake ($N_{mCherry}$=11, N_{hM4Di}=17, Two-way RM ANOVA, Day x Group interaction, F(2,52)=0.5835, p=0.5615). Data are presented as mean values + SEM. All statistical tests were performed two-sided. Source data are provided as a Source Data file.

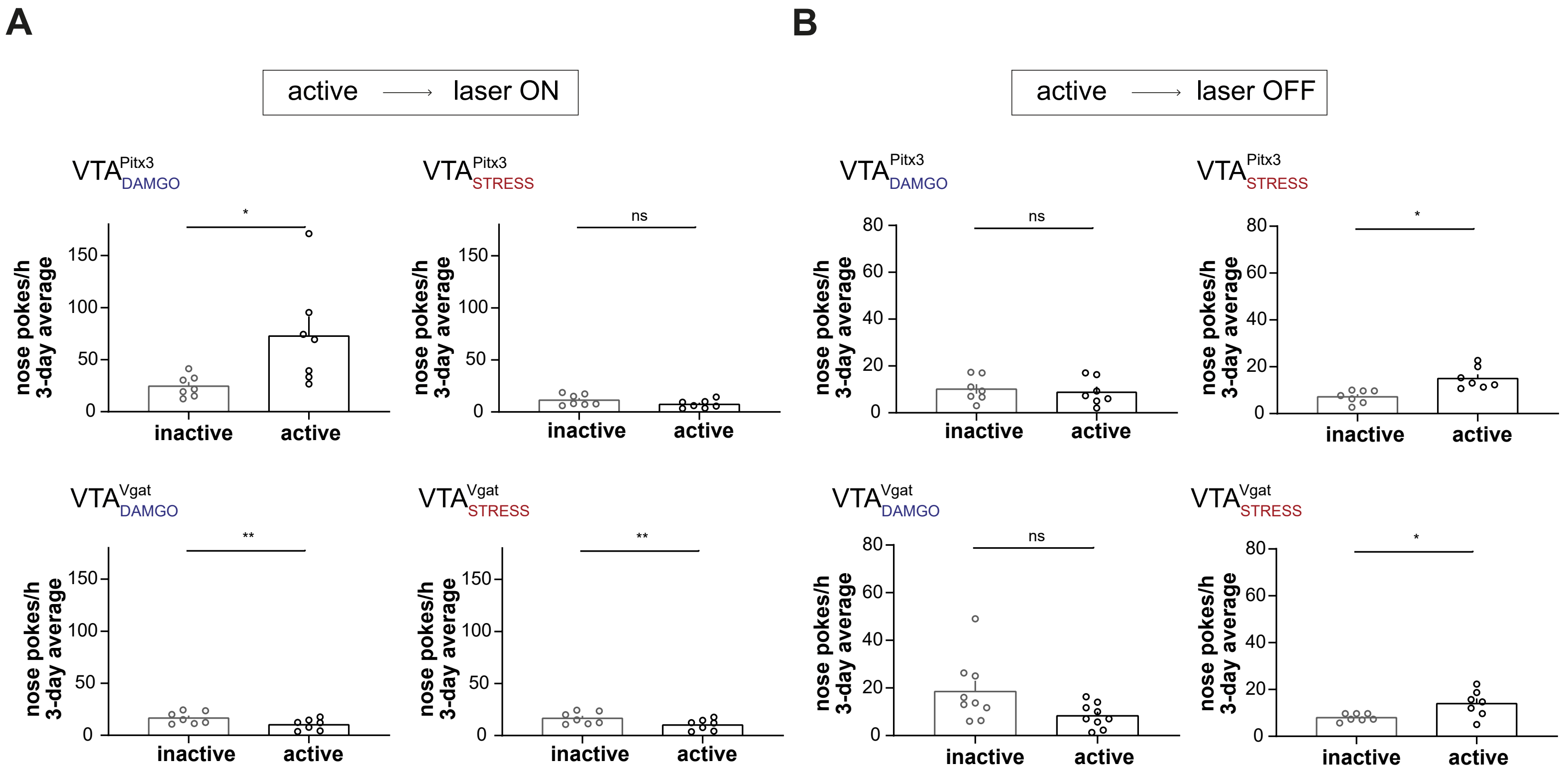

Supplementary Figure 6

SUPPLEMENTARY FIGURE 6 | Control measures for intersectional manipulation of neurotransmitter-defined subsets of VTA_{DAMGO} or VTA_{STRESS} ensembles. (A) Top: The 3-day average number of nose-pokes to activate a 473nm laser for activation of the dopaminergic part of VTA_{DAMGO} and VTA_{STRESS} groups (VTA_{DAMGO} : N=7, paired t test, t(6)=2.707, p=0.0352. VTA_{STRESS}: N=7, paired t test, t(6)=2.049, p=0.0864). Bottom: The 3-day average number of nose-pokes to activate a 473nm laser for the GABAergic part of VTA_{DAMGO} and VTA_{STRESS} groups (VTA_{DAMGO} : N=9, paired t test, t(8)=4.412, p=0.0022. VTA_{STRESS}: N=7, paired t test, t(6)=5.728, p=0.0012). **(B)** Top: The 3-day average number of nose-pokes to deactivate a 473nm laser for the dopaminergic part of VTA_{DAMGO} and VTA_{STRESS} groups (VTA_{DAMGO} : N=7, paired t test, t(6)=0.6896, p=0.5162. VTA_{STRESS}: N=7, paired t test, t(6)=3.196, p=0.0187). Bottom: The 3-day average number of nose-pokes to deactivate a 473nm laser for the GABAergic part of VTA_{DAMGO} and VTA_{STRESS} ensembles (VTA_{DAMGO} : N=9, paired t test, t(8) = 1.995, p=0.0811. VTA_{STRESS}: N=7, paired t test, t(6)=3.045, p=0.0227). Data are presented as mean values + SEM. All statistical tests were performed two-sided. Source data are provided as a Source Data file.
