# Supplementary material for: Distinct ventral tegmental area neuronal ensembles are indispensable for reward-driven approach and stress-driven avoidance behaviors
Source: Nat Commun. 2025 Apr 2;16:3147. doi: 10.1038/s41467-025-58384-3 (PMC11965480; doi:10.1038/s41467-025-58384-3)
Supplement: Supplementary file 2 — Description of Additional Supplementary Files [file 41467_2025_58384_MOESM2_ESM.pdf]

Description of Additional Supplementary Information

Supplementary Data 1

Description: Source Data for statistical analyses.

Supplementary Data 2

Description: Statistical analysis outcomes.
